# Utilization of Artificial Intelligence (AI) in Healthcare Decision-Making Processes: Perceptions of Caregivers in Saudi Arabia

**DOI:** 10.7759/cureus.67584

**Published:** 2024-08-23

**Authors:** Horaya A Amin, Turki M Alanzi

**Affiliations:** 1 Public Health, Imam Abdulrahman Bin Faisal University, Dammam, SAU; 2 Health Information Management and Technology, Imam Abdulrahman Bin Faisal University, Dammam, SAU

**Keywords:** providers, saudi arabia, decision-making, healthcare, artificial intelligence, utilization

## Abstract

Background

In the evolving landscape of healthcare, artificial intelligence (AI) has emerged as a transformative force, revolutionizing decision-making processes. Through advanced machine learning and data analytics, AI promises precision and personalized treatments, particularly impactful in diagnostics and personalized medicine.

Aim and objectives

This study aims to investigate the utilization and effectiveness of AI algorithms among healthcare caregivers, focusing on decision-making processes. Objectives include assessing AI adoption prevalence, understanding demographic factors influencing utilization, and evaluating its impact on decision-making dynamics, diagnostics, and personalized medicine.

Methods

Employing a quantitative cross-sectional approach, an online questionnaire was distributed to 224 healthcare professionals. The survey covered AI familiarity, perceived effectiveness, and potential barriers. Data analysis utilized descriptive statistics and bivariate analyses.

Results

Seventy-five percent of caregivers reported that they used AI in the decision-making process, with nurses representing a significant majority (50.4%). Bivariate analyses identified correlations between AI utilization and demographic variables, emphasizing its diverse adoption across specialties.

Conclusion

This study reveals substantial AI adoption, notably among nurses, indicating a transformative shift in decision-making processes. The findings underscore AI's potential in diagnostics and personalized medicine, highlighting the need for targeted interventions and collaborative efforts to address challenges and maximize AI benefits in healthcare.

## Introduction

In the ever-evolving landscape of healthcare, artificial intelligence (AI) stands as a beacon of innovation, fundamentally reshaping decision-making processes across the entire spectrum of medical practice [1]. Through the sophisticated integration of advanced machine learning algorithms and data analytics, AI has ushered in a paradigm shift, promising unprecedented precision, individualized treatments, and an unparalleled standard of patient care [2].

AI's impact is most pronounced in the domain of diagnostics, where its algorithms, honed through extensive datasets, exhibit a remarkable ability to decipher intricate medical images with a level of accuracy that surpasses human capability [3]. This transformative power has redefined disease detection, particularly in challenging cases like cancer and cardiovascular ailments [4]. Moreover, AI's predictive prowess extends to proactive disease management, analyzing historical patient data to identify high-risk individuals and enabling timely interventions that substantially enhance patient outcomes [4].

Beyond diagnostics, AI-driven systems are pioneering the era of personalized medicine. By meticulously analyzing diverse datasets encompassing genomics and clinical records, AI tailors treatment regimens to individual patients’ genetic predispositions, medical histories, and lifestyles [5]. This personalized approach not only maximizes treatment efficacy but also minimizes adverse effects, heralding a new era of healthcare that revolves around the unique needs of each patient [6].

Furthermore, AI optimizes the operational backbone of healthcare institutions. Streamlining tasks ranging from staff scheduling to inventory management, AI ensures the seamless functioning of medical facilities, optimizing resource allocation and enhancing overall efficiency [6]. However, this transformative journey is not without hurdles. Ethical considerations, including safeguarding patient privacy and addressing biases in algorithms, demand meticulous attention [7]. Regulatory frameworks must evolve to strike a delicate balance between fostering innovation and upholding ethical standards, ensuring responsible AI deployment in healthcare [7].

As healthcare professionals, researchers, and policymakers collaborate to navigate these challenges, the integration of AI becomes not merely a technological advancement but a cornerstone of a patient-centered healthcare future [8]. Through collective efforts, these challenges can be surmounted, enabling the healthcare sector to fully harness AI's potential [8]. This integration promises a future where medical decisions are not just precise and efficient but also deeply human, ensuring that every patient receives personalized, compassionate, and optimal care [5].

## Materials and methods

Study setting

This study was focused on healthcare professionals in various hospitals and clinics across Saudi Arabia. The study utilized a quantitative survey methodology, distributing questionnaires electronically through social media applications widely used in the healthcare community.

Target population and sample size

This study targeted all Saudi and non-Saudi caregivers, including doctors, nurses, and administrators, who were actively engaged in decision-making processes. The purpose was to analyze the utilization of AI technologies in healthcare decision-making processes through a structured online questionnaire. Individual questionnaires were excluded from the study if major sections of key variables were incomplete or if they were completed by non-caregivers. The sample size was 224 caregivers, calculated using a finite population correction formula. Participants were selected through convenience sampling, considering their availability and willingness to participate. The survey aimed to gather diverse representations from different medical specialties and healthcare facilities.

Research approach and data collection

The study applied a quantitative cross-sectional approach, as the data were collected at one point in time using a questionnaire. In this study, the cross-sectional method was used due to time constraints and because it is often used to look at the relationships between independent variables and the desired outcome. This study started in October 2023 and concluded in December 2023.

An online-based questionnaire was distributed using a convenience sampling technique. The snowball method was used to facilitate the distribution of the questionnaire. The use of online questionnaires offers unparalleled convenience, allowing researchers and participants to engage from anywhere in the world. They streamline data collection, reducing manual errors and ensuring prompt analysis. Paper questionnaires require extensive time and resources for distribution and collection. They have limited geographical reach, potentially leading to biased samples. Lack of interactivity can result in disengaged participants and incomplete responses. Manual data entry is labor-intensive and error-prone. Physical documents pose security risks and ethical concerns regarding confidentiality. Compared to online surveys, paper questionnaires lack efficiency, adaptability, and data security [9].

The questionnaire, designed specifically for this study, consisted of a five-point Likert scale ranging from “strongly disagree” (1) to “strongly agree” (5), with questions assessing participants' familiarity with AI applications in healthcare, their perceptions of AI's effectiveness, and potential barriers faced in its implementation. The survey was distributed using popular social media platforms, such as LinkedIn and Twitter, ensuring broad access to healthcare professionals. Internal consistency and reliability of the scale were tested using the standard Cronbach’s α, which was developed by George and Mallery, who found that the instrument was reliable and excellent (overall Cronbach’s α = 0.881). The process of validating and developing an instrument largely focuses on reducing errors in the measurement process [10]. Expert review was conducted to ensure the face and content validity of the questionnaire. Validation increases the data accuracy. The questionnaire was sent to a panel of experts, including experts on survey development, health researchers, and study participant representatives.

The questionnaire consisted of 17 questions. The first question was about using AI in healthcare decision-making, and the other questions were designed to collect information about the perspectives of caregivers. It was divided into two domains: the first (four items) addressed the utilization of AI, and the second (six items) addressed the effectiveness’ of AI in medical diagnosis and treatment planning. There were six supplementary demographic-related questions.

Ethical approval

Imam Abdulrahman Bin Faisal University’s Institutional Review Board (IRB) granted ethical approval (IRB-PGS-2023-03-569). Informed consent was obtained from participants using the following statement: “Your response is completely anonymous, and there will be no names or e-mail addresses. You can be sure that all information collected in this study will be kept strictly confidential. Only aggregated data will be presented in any reports of findings of this study.”

Data analysis

The data were entered into Microsoft Excel (Microsoft® Corp., Redmond, WA) and then translated into SPSS (IBM SPSS Statistics for Windows, IBM Corp., Armonk, NY) to ensure that no data were missing or inaccurate before the data analysis. The data analysis included the use of descriptive univariate statistics, frequencies, and percentages to analyze the demographic data. Measures of central tendency were used for the continuous variables in the study. The Skewness and Kurtosis parameters in SPSS were used to check the normality. Likert scale responses were converted into numerical values for quantitative analysis; t-test and ANOVA were performed to identify correlations between variables, such as AI familiarity and perceived effectiveness.

## Results

Demographic characteristics of the caregivers

A total of 224 caregivers were included in the study (Table 1). Only 186 (75%) caregivers reported that they used AI in the decision-making process, while 56 (25%) caregivers reported that they were not using AI in the design-making process. Most of the caregivers were male (n = 150; 67%), and there were 74 (33.3%) female caregivers. The age group with the highest percentage of caregivers (45%) was 25 years old or younger, while the age group with the lowest percentage of caregivers (6%) was 41 to 45 years old. More than half of the caregivers who participated were nurses (n = 113; 50.4%), while less than 6.7% of those who participated were residents. The majority of the caregivers who completed the questionnaire were Saudi citizens (173.7%). Most of the caregivers completed their medical training in Saudi Arabia (n = 146; 65.2%), and 77 (34.4%) caregivers completed their training in other countries. Regarding years of working experience, 104 (46.4%) caregivers had less than one year of experience, and 30 (13.4%) had more than 10 years of experience.

**Table 1:** Demographic characteristics

Demographic data	Descriptive statistics, n (%)
AI utilization	
Yes	168 (75)
No	56 (25)
Gender	
Male	150 (67)
Female	74 (33)
Age	
25 years old or younger	101 (45)
26 to 30 years old	25 (11)
31 to 35 years old	18 (8)
36 to 40 years old	23 (10)
41 to 45 years old	15 (6)
46 to 50 years old	19 (8)
51 years old or older	23 (10)
Nationality	
Saudi	173 (88.2)
Non-Saudi	51 (22.8)
Specialty	
Consultant	33 (14.7)
Nurse	113 (50.4)
Resident	15 (6.7)
Intern	17 (7.6)
Specialist	36 (16.1)
Other	10 (4)
Work experience	
Less than one year	104 (46.4)
One year and less than five years	54 (24.1)
Five years and less than 10 years	36 (16.1)
10 years and above	30 (13.4)
Completing medical training	
Saudi Arabia	146 (65.2)
Other country	77 (34.4)

Utilization and effectiveness of AI algorithms

Each variable was assessed using a set of questions (11 items), and each respondent was assigned a score for each question. These scores were added together to create an overall score for each variable. The overall scale for each variable is the average of this total score. The normality test was applied, and the results showed that the utilization score and effectiveness score were normally distributed; the mean of the utilization was 7.70 with a standard deviation (SD) of 4.440, and the mean of effectiveness was 9.79 with an SD of 5.836 (Table 2). The relationships between using AI and demographic variables (gender, age, nationality, specialty, training courses, work experience, and fellowship country) were tested using the t-test and ANOVA test (Table 2). All the variables were statistically significant (age: p > 0.01; nationality: p > 0.002; specialty = p > 0.001; training, work experience: p > 0.001; fellowship country: p > 0.001). However, age was statistically not significant (p < 0.001) (Table 2).

**Table 2:** Bivariate analysis of factors in the study Note: *p-value < 0.05

Demographic data	Utilization score mean (SD)	Test statistics (p-value)	Effectiveness score mean (SD)	Test statistics (p-value)
AI utilization	-	t = -22.05 (0.001*)	-	t = -22.91 (0.007*)
Yes	5.58 (2.16)	-	6.98 (2.914)	-
No	14.05 (3.28)	-	18.25 (3.905)	-
Gender	-	t = -4.447 (0.667)		t = -4.166 (0.396)
Male	6.81 (4.324)	-	8.69 (5.638)	-
Female	9.50 (4.139)	-	12.03 (5.623)	-
Age	-	f = 58.63 (0.001)*	-	f = 53.62 (0.001*)
25 years old or younger	4.55 (1.539)	-	5.75 (2.027)	-
26 to 30 years old	7.44 (2.485)	-	9.92 (3.989)	-
31 to 35 years old	8.61 (4.327)	-	11.22 (6.160)	-
36 to 40 years old	7.74 (1.71)	-	8.91 (2.827)	-
41 to 45 years old	12.20 (3.72)	-	16.27 (4.949)	-
46 to 50 years old	12.26 (4.13)	-	16.21 (5.319)	-
51 years old or older	14.30 (4.30)	-	17.65 (5.193)	-
Nationality	-	t = -6.59 (0.012*)	-	t = -5.702 (0.002*)
Saudi	6.72 (3.97)	-	8.66 (5.287)	-
Non-Saudi	11.00 (4.38)	-	13.63 (6.026)	-
Specialty	-	f = 5.629 (0.001*)	-	f = 5.812 (0.001*)
Consultant	8.24 (3.052)	-	10.58 (4.465)	-
Nurse	6.31 (4.631)	-	7.98 (5.727)	-
Resident	8.27 (3.595)	-	10.67 (5.260)	-
Intern	8.76 (2.587)	-	10.35 (3.316)	-
Specialist	10.19 (4.59)	-	12.94 (6.423)	-
Other	9.22 (3.563)	-	13.44 (5.981)	-
Work experience	-	f = 100.8 (0.001*)	-	f = 87.107 (0.001*)
Less than one year	4.70 (1.832)	-	6.01 (2.514)	-
One year and less than five years	7.69 (2.370)	-	9.72 (3.969)	-
Five years and less than 10 years	11.11 (4.18)	-	14.08 (6.007)	-
10 years and above	14.00 (4.52)	-	17.90 (4.992)	-
Completing medical training	-	t = -8.15 (0.001*)	-	t = -7.59 (0.001*)
Saudi Arabia	6.16 (3.626)	-	7.89 (4.840)	-
Other country	10.65 (4.38)	-	13.47 (5.857)	-

## Discussion

The primary objective of this study was to delve into the usage and effectiveness of AI algorithms within caregivers' decision-making processes. An exploration of the demographic characteristics of the participants yielded valuable insights, shedding light on the composition of our study sample. Among the 224 caregivers considered, a significant majority (75%) reported integrating AI into their decision-making processes, while the remaining 25% did not. This points to a substantial adoption of AI technologies in the healthcare decision-making landscape, underscoring the growing integration of AI into medical practices.

An analysis of the gender distribution among caregivers in our study revealed a predominant representation of males (67%). This finding aligns with previous research that has documented a relationship between participant gender and technology use [11]. Notably, studies, such as those by Dyck et al. [11], Venkatesh et al., and Chinyamurindi et al. [12-13], found that women often reported difficulty and discomfort when using technology, contrasting with men who found it pleasant, easy, and useful. Additional research has suggested that women are less likely to adopt and use technology compared to men [14]. These findings indicate a resistance among women, relative to men, in engaging with and adopting new technologies and pursuing future careers in technology.

The literature provides conflicting perspectives on the effects of age and skills. As noted by Dyck et al. [11], older individuals tend to harbor more negative attitudes toward technology compared to their younger counterparts. This observation resonates with our study, where caregivers aged 25 years or younger constituted the largest proportion (45%) of participants, highlighting the significant presence of younger healthcare professionals in our investigation. This emphasizes the importance of understanding the viewpoints of younger generations within the healthcare workforce concerning AI adoption, as they may contribute unique insights and expectations.

The findings of this research unveil intriguing insights into the utilization of AI among caregivers, particularly the striking prevalence of AI adoption among nurses, accounting for 50.4% of the study participants - a finding similar to that of a previous study [12]. The substantial representation of nurses employing AI in their decision-making processes highlights the pivotal role technology plays in the nursing profession [14]. The implications of this trend extend beyond mere statistics, suggesting that nurses, as frontline healthcare providers, recognize the tangible benefits AI brings to patient care and clinical workflows. The emphasis on patient-centric care, streamlined workflows, and efficient clinical decision support elucidates the potential of AI to enhance healthcare delivery. Moreover, the higher adoption rates among nurses prompt further inquiry into the specific applications embraced and the perceived impact on their decision-making dynamics [15]. This study underscores the need for targeted interventions and training programs that cater to the evolving technological landscape in healthcare, ensuring that caregivers across diverse specialties can harness the full potential of AI for improved patient outcomes and enhanced healthcare practices. Additionally, future research could explore the nuanced factors contributing to the varying adoption rates among different caregiver categories, shedding light on the intricacies of integrating AI into diverse healthcare roles.

Despite the strengths of this study, such as the high reliability of the collected data, it is essential to acknowledge notable limitations, particularly concerning sample size and representativeness. To enhance future studies in this vein, it is imperative to include a more extensive and diverse sample, ensuring active participation from elderly individuals and maintaining a balanced representation of both male and female participants. Nevertheless, these limitations do not diminish the robustness of the study's findings.

## Conclusions

In conclusion, this research illuminates the transformative impact of AI on healthcare decision-making. The study's findings underscore a significant adoption of AI technologies among caregivers, with 75% incorporating AI into their decision-making processes. Notably, nurses emerge as the forefront adopters, constituting 50.4% of participants, showcasing the pivotal role of technology in the nursing profession. The prevalence of AI adoption among younger healthcare professionals, as evidenced by 45% of participants aged 25 years or younger, signals a shift in generational perspectives toward embracing technological innovations.

The study highlights AI's influence in diagnostics, where advanced algorithms exhibit superior accuracy in deciphering complex medical images and redefining disease detection, especially in challenging cases such as cancer and cardiovascular ailments. Beyond diagnostics, AI's capacity for personalized medicine is emphasized, tailoring treatment regimens based on individual genetic predispositions, medical histories, and lifestyles. This personalized approach not only maximizes treatment efficacy but also minimizes adverse effects, marking a paradigm shift toward patient-centric care.
